# Maximization delays decision-making in acute care nursing

**DOI:** 10.1038/s41598-024-56037-x

**Published:** 2024-03-06

**Authors:** Ricardo Tejeiro, Antonio Romero-Moreno, Alberto Paramio, Serafín Cruces-Montes, María Concepción Galán-Artímez, Judit Santos-Marroquín

**Affiliations:** 1 Department of Psychology, Liverpool John Moores University, Liverpool, UK; 2 Instituto Universitario para el Desarrollo Social Sostenible (INDESS), Universidad de Cádiz, Cádiz, Spain; 3 University Hospitals Birmingham NHS Foundation Trust, Birmingham, UK

**Keywords:** Maximization, Nursing, Decision-making, Practice-primed decision model, Acute care, Psychology, Human behaviour

## Abstract

The maximization personality trait refers to the tendency to face decision-making situations along a continuum from exhaustively analysing all the options (maximize) to choosing the one that exceeds a subjective threshold of acceptability (satisfy). Research has revealed the influence of maximizing on decision making, although little is known about its possible role in high risk and high uncertainty situations. A sample of 153 active Spanish nurses, with an average experience of 11 years, completed a maximization questionnaire and responded to written vignettes depicting time-demanding decision making in which three options were offered, representing delayed action, non-action, and immediate action. Two vignettes presented critical situations related to acute care during the COVID-19 pandemic, whilst two vignettes presented non-nursing scenarios. People high in maximization took longer to choose and were more likely to choose non-action. No relationship was found between maximization score and the subjective experience of the person making the choice. Maximization had no significant correlation with years of experience nor perceived expertise. Greater perceived expertise was associated with lower indecision and greater confidence. When participants answered nursing vignettes, they took longer to respond, but chose less delayed action and more immediate action. Our results suggest that maximization plays only a relative role in acute care decision-making in nursing, as compared to contextual variables and expertise. They also support a domain general approach to this personality trait. Findings are consistent with Nibbelink and Reed's Practice-Primed Decision Model for nursing.

## Introduction

“*We have felt helpless, with a brutal sense of uncertainty and the pressure of having to learn and decide on the fly*”.

Aroa López-Martín, A&E Head Nurse at Vall d'Hebron Hospital (Barcelona, Spain), 16 July 2020.

Nurses are the largest group of serving staff in health service organizations^1^. The dynamic and uncertain nature of health care environment requires nurses to be competent decision makers in order to respond to clients' needs. In other words, “they should be able to sift and synthesize information, make decisions and appropriately implement these decisions to solve their clients' problems in the context of a multidisciplinary team”^2^.

The literature on decision-making in nursing has explored a variety of factors including intuition, cognitive bias, thinking processes, information seeking, knowledge structure, professional values, and accountability, amongst others^3–5^. It has also frequently drawn from non-nursing theories, like the Cognitive Continuum Theory^6^, whose application to nursing was tested by Cader et al.^7^. However, theoretical models focused on decision making in nursing are surprisingly rare, and some of the proposals (e.g., the four decision-making models identified by Kosicka et al.^8^), could be seen as referring to decision-making styles rather than models.

A noteworthy step in this direction is Nibbelink and Reed’s^9^ Practice-Primed Decision Model (PPDM). The PPDM is based on Klein's^10^ Recognition-Primed Decision (RPD) Model, which states that when people face difficult decisions under time pressure, they compare the situation with previously generated patterns based on their practical experience and knowledge, a 'pattern matching' process that is complemented with 'mental simulation'. Based on the RPD, the Naturalistic Decision Making (NDM; Klein et al.^11^) Model incorporates elements such as ill-structured problems, uncertain dynamic environments, ill-defined and changing goals, feedback loops, time constraints, high stakes, multiple decision makers, and organisational norms and goals. After a review of the literature^12^, the PPDM develops this view by suggesting that the decision-making process in nursing is initiated by a situation awareness factor, defined as “the perception of the elements in the environment in a volume of time and space, the comprehension of their meaning and the projection of their status in the near future”^13^. Six additional factors influence decision according to the PPDM—experience, nursing unit culture, education, autonomy, collaboration with colleagues, and bias resulting from the use of heuristics^14^.

Although decision-making by nurses is usually grouped under the term 'clinical judgment', in practice it can vary significantly between the different settings^15^. The PPDM was presented in the context of research on decision-making in acute care nursing and responds to the peculiarities of this setting—such as the need to consider numerous potentially conflicting factors^16^ and make numerous decisions under temporal pressure. Situations like the COVID-19 pandemic add elements to the equation, such as the relocation of nurses to wards that are outside their field of expertise^17^, perception of physicians’ lack of knowledge, precariousness of the facilities, or lack of information about the patients as they were admitted without accompanying relatives^18^.

The PPDM, however, does not consider other psychological variables that the literature associates with decision making. One such psychological variable is the maximization construct. This construct is based on Simon's^19^ suggestion that in most cases, our cognitive limitations prevent us from optimising choices (maximization) and therefore we limit ourselves to “satisfying” or evaluating options until we find one that suits us. From this formulation, Schwartz et al.^20^ propose maximization as a one-dimensional trait at whose extremes are the so-called "maximizers" and "satisfiers".

Although various theoretical and practical approaches to the concept of maximization have been proposed^21^, in the last two decades numerous studies have illustrated its possible impact on decision-making behaviour. Thus, it has been reported that maximizers prefer to face decisions involving a large number of alternatives, use a rational-cognitive style for decision making, and ruminate more than satisficers on the choice after it has been made (see Shortland et al.^22^ for a review). Notably, those who consistently try to maximize their decisions have a greater tendency to procrastinate^23^ and to fall into decisional inertia or avoidance, redundant deliberation, or failure to implement a decision^24,25^.

Studies on the role of maximization in decision making have typically been conducted in the context of consumer behaviour^26,27^. However, most decisions in these contexts are relatively mundane in nature and differ substantially from those made in high stakes situations, which involve uncertainty, or are subject to time pressure^28^. These types of situations have received much less attention from researchers. The few studies available on military personnel and on police officers^22^ reveal that in these scenarios, maximizers tend to be slower in decision making and perceive the process as more difficult. To the best of our knowledge, no study has been conducted on the impact of maximization on the nurses’ decision-making process.

It is interesting to note that the relative importance of this gap depends on the perspective one adopts. Historically, it has been generally assumed that maximization has a domain general effect on decision making, which is supported by findings of similar execution across various domains^29^. Some authors, however, suggest a situational approach, with different context-dependent manifestations of maximization^30^, or different levels of maximization depending on the relative value that each domain has for the person making the choice^31^. In order to check if the effect of maximization in our study is context-dependent, we decided to compare our participants’ responses to decision-making situations both related and unrelated to their usual work context as acute care nurses.

With the above in mind, the present study tries to answer the question ‘what is the role that the trait maximization plays on nurse decision making in acute care?’ In the same way as other researchers on maximization (e.g., Shortland et al.^22^), we differentiated two types of variables in relation to decision-making—observable behavioural variables and associated subjective experience. Observable behavioural variables indicating avoidance were slower decision time, higher number of ‘delayed action’ and ‘non-action’ selections, and lower number of ‘immediate action’ options (see variables below). Subjective experience indicating avoidance were higher perceived difficulty, and lower confidence in one’s choices. From this, our working hypotheses are: (H1) Greater maximization scores will be associated with higher likelihood to make avoidant behavioural choices, (H2) Greater maximization scores will be associated with higher subjective difficulty, and (H3) The effect of maximization on decision making will not vary across domains.

Additionally, experts have been consistently found to engage in more selective attention and rely on heuristics to make decisions^32^. Experienced nurses, in particular, have been found to employ intuition in their decision making whereas inexperienced nurses use an analytic approach with careful consideration of all available information^33^. Whether this should be considered as an advantage—as experts are more able to recognise situations and rapidly generate good solutions^10^—or as a source of bias^34^, it seems logical to conclude that the tendency to maximize will decrease as the individual’s expertise increases. From this, we add the hypotheses that individuals with greater perceived expertise will have (H4) lower maximization scores, (H5) less tendency to make avoidant behavioural choices, and (H6) less tendency to report subjective experience indicating avoidance.

## Materials and methods

### Participants

The sample for this study included 153 nurses registered in the Nursing Professional Society of Cadiz (NPSC), in Spain; 84.97% were female, with 1–42 years of professional experience (*M* = 10.86, *SD* = 10.46). The male/female ratio is very similar to that of the nursing population in Spain (84.3% female; INE, 2019) and only slightly below the world average (90%; WHO, 2020). The only inclusion criterion was being an active registered nurse in the NPSC, which implies the exclusion of vulnerable individuals as defined by the Code of Ethics of our universities. Recruitment of participants was facilitated by the NPSC by posting the announcement on their internal web site.

### Materials

A survey containing the following elements was prepared on the online platform Qualtrics.

#### Demographic details

These include self-described gender, number of years of practice as a nurse, and self-perceived level of expertise in nursing. Following Verster et al.’s^35^ suggestion, a five-point Likert-type response from 0 = almost none, to 5 = skilled expert was used for the latest, as no brief scales exist that assess perceived nursing expertise in a single dimension due to the difficulty in defining the concept^36^.

#### Maximization

Maximization was measured with Dalal et al.^37^ MTS-7, a 7-item questionnaire with a 5-point Likert-type response for-mat from 1 = completely disagree to 5 = completely agree. The scale includes statements such as “I don’t like having to settle for good enough” and “I will wait for the best option, no matter how long it takes”. The overall score is the result of the addition of the item scores. The original authors reported a one-factor structure and a coefficient alpha of 0.82. A validated version of the MTS-7 with α = 0.78 was used (Tejeiro et al., in preparation).

#### Decision making

The research procedure (see below) allows for the calculation of two dependent measures from the pattern of choices made by respondents: (1) Decision Time (DT), or the overall time it takes the participant to choose a course of action–time they needed to submit the desired option, and (2) Type of decision (DEC): Delayed action, non-decision, or immediate action. In addition, after each vignette participants are asked to indicate, on a 5-point Likert-type response format, the perceived degree of difficulty (DIF) as well as their degree of confidence in the action selected (CON).

### Ethics

The researchers obtained the approval of the Ethical Board of the University of Liverpool (approval No. 9834). The selection of characters for the vignettes intended to minimise the possibility of bias—the six surnames used are amongst the 20 most frequent surnames in Spain^38^, and the eight characters about which participants are requested to decide are equally divided between males and females. The study was conducted in compliance with the Declaration of Helsinki of 2013 (Seventh revision, 64th Meeting, Fortaleza) and the Spanish Organic Law 3/2018, of December 5, Protection of Personal Data and Guarantee of Digital Rights in accordance with the Regulation (EU) 2016/679 of the European Parliament and of the Council, of 27 April 2016. Participants did not receive any reward for participating in the study. An informed consent text was sent to all participants together with the questionnaire. The participants were guaranteed confidentiality and anonymity.

### Procedure

An announcement was posted on the NPSC internal web site summarising the object and main features of the research, offering the researchers’ emails for requests of further information, and inviting interested people to click on a link which will lead them to the online survey in Qualtrics. The survey was preceded by the participant information sheet and the consent form. Participants who ticked all acceptance boxes in the latter were allowed to proceed to the survey items as presented in “Materials” above, followed by a short debriefing statement. Those who refused or fail to tick all acceptance boxes were not allowed to access the survey; they were thanked for their time and excluded from the study. The recruitment and data collection period began in January 2022 and ended in March of the same year. Participants needed about 20–25 min to complete the survey, including vignettes.

#### Decision making task

The decision-making task consists of four immersive desk-based vignettes that force the individual to decide under conditions of uncertainty. Best practice in the design and implementation of vignettes was followed, including those situations must appear plausible and real to participants; they contain sufficient context for respondents to have an understanding but are vague enough to leave participants to define the situation in their own terms; and they are internally consistent and not too complex^39^.

The set includes two non-nursing vignettes developed by the research team based on their experience on decision making, and two nursing vignettes directly related to COVID-19 patients in acute care settings. These vignettes were developed by one of the researchers based on their experience as a nurse and through discussions with expert colleagues. All vignettes follow the same format. Participants are presented with a context page, after which they are requested to press ‘next’ when they are happy that they understand the situation*.* The second page (incident) adds a problem situation (again asking the participant to press ‘next’ when they are happy that they understand it)*.* The third page asks the participant to select one of three courses of action (3-alternative forced choice). One of the choices represents an *immediate action* (e.g., “you call an ambulance”); another choice invites the participant to wait until a potentially key piece of information is available, or *delayed action* (e.g., “You remain with Miss Perez to check on her progress and, if there is no further worsening in the next hour or so, continue your visits.”); the remaining choice represents the refusal to take any action independently, or *non-action* (e.g., “You remain with Miss Perez until your supervisor is available no matter how long it takes, though that implies missing your next visits”). The order of the three types of choices was randomised across the vignettes. It should be noted that we do not purport that any option is the right or the most appropriate one, as our focus was to analyse our participants’ decision-making behaviour as a function of the independent variables—not whether the action was correct or incorrect. ESM Appendix A presents each vignette’s context and incident pages.

### Analysis

The SPSS version 29 statistical package with the AMOS module version 27 was utilised for data analyses. These include descriptive statistics of overall performance, and Confirmatory Factor Analysis (CFA) of the MTS-7. We are aware that our sample size was smaller than the 200 participants recommended by most, but we met the 10:1 ratio of respondents to variables generally accepted^40^. The fit of the data in CFA was tested with absolute fix indices (normed Chi^2^, χ^2^/df; and Standardized Root Mean Square Residual [SRMR]), parsimony-adjusted indices (Steiger-Lind Root Mean Square Error of Approximation [RMSEA]), and incremental fit indices (Tucker-Lewis index [TLI]; and Bentler Comparative Fit Index [CFI]). χ^2^/df values between 1 and 2 are indicative of a good fit and between 2 and 3 an acceptable model fit^41^. Values < 0.08 in the SRMR, ≤ 0.06 in the RMSEA^42^, > 0.90 in the TLI^43^ and ≥ 0.95 in the CFI^42^ are representative of a good model fit.

Multi-level modelling (MLM) tested the effect of maximization on each dependent variable while controlling for professional experience and vignette type. Four vignettes and 149 participants results in a total of 596 expected data points (decision-making trials) per dependent variable. As all participants received all vignettes (fully crossed design), the multilevel models were fitted with random intercepts for the participants and the vignettes, which accounts for between-vignette heterogeneity and intra-respondent correlation. None of the two conditions for an alternative nested design—that each participant receives a unique set of vignettes or the dimensions defining the vignettes exhaust the variability in the vignette universe^44^—were met. Normality was analysed using the Shapiro-Wilks tests, given the moderate sample size. Differences in performance across vignettes were measured with Chi-square and Mann–Whitney’s U tests; effect size is measured with Cramer’s V and *r*.

## Results

### Descriptive statistics

#### Overall performance

Participants took a Median of 25.12 s to submit a decision (IQR = 22.12, Min = 2.46, Max = 155.25); however, responses to nursing vignettes (Mdn = 30.96, IQR = 24.55, Min = 4.67, Max = 155.25) took significantly longer than responses to non-nursing vignettes (Mdn = 22.58, IQR = 18.12, Min = 4.67, Max = 130.23) (U = 23,848, *p* < 0.001, *r* = 0.28). On average, participants scored the vignettes as medium difficulty (Mdn = 3, IQR = 1, Min = 1, Max = 5), and felt medium to high confidence in their decision (Mdn = 4, IQR = 1, Min = 1, Max = 5); no significant differences were found by type of vignette.

Fifty-eight percent of decisions corresponded to the *immediate action* option, 19% to the *non-action* option, and 22.9% to *delayed action.* Table 1 shows the distribution of response type by type of vignette (nursing vs non-nursing). Chi-square analyses reveal significant differences between types of vignette (χ^2^_(2)_ = 16.47, *p* < 0.001, Cramer’s V = 0.18), with greater tendency to delayed action in non-nursing vignettes and to immediate action in nursing vignettes. All variables were non-normally distributed (Shapiro-Wilks tests, *p* > 0.001).

**Table 1:** Response distribution by type of vignette; n(%).

Response	Vignette type		
	Total	Non-nursing	Nursing
Delayed	123 (22.9)	87 (28.9)	36 (15.3)
Non-action	102 (19)	60 (19.9)	42 (17.9)
Immediate	311 (58)	154 (51.2)	157 (66.8)
Total	536 (100)	301 (100)	235 (100)

#### Maximization

Overall, participants’ Maximization scores ranged from 10 to 34 (Mdn = 27, IQR = 6). Maximization had no significant correlation with years of experience (rho = 0.07 *p* = 0.180) or perceived expertise (rho = 0.05 *p* = 0.342). The internal consistency of the MTS scale was high (Cronbach’s alpha = 0.84) and similar to that reported by the scale’s authors; the reliability of the scale does not increase when any of the items is deleted. All corrected item-total correlations were higher than 0.45. In CFA, the overall fit of the one-factor model was good for all indices (χ^2^/df = 1.69, CFI = 0.99, TLI = 0.99, SRMR = 0.02, RMSEA = 0.04 [CL90 0.00–0.07]).

### Data modelling

For each dependent variable (decision making), the combination of four vignettes and 153 participants yielded 612 data points; early dropout of participants resulted in 536 data points. Multi-level modelling (MLM) for each of the dependent variables organised these data points both by the random effects of the vignettes and by participants. From this structure, a two-level MLM was used to estimate the main effect of maximization on the decision-making variables (see Table 2), controlling for perceived expertise, gender, and vignette type. Years of experience was not included in the models due to its high correlation with perceived expertise (Spearman’s rho = 0.60, *p* < 0.001), to avoid multicollinearity.

**Table 2:** Multilevel linear regressions for decision making scorings with crossed-random effects.

Models	β	SE	*p*
(1) Decision time			
Constant	19.474	7.835	0.013
Maximization	0.445	0.209	0.034
Perceived expertise	− 1.048	4.552	0.500
Gender (Female = 1)	− 0.180	2.787	0.949
Nursing vignette (Yes = 1)	10.509	1.986	< 0.001
(2) Difficulty			
Constant	3.406	0.390	< 0.001
Maximization	− 0.010	0.010	0.330
Perceived expertise	0.061	0.077	0.428
Gender (Female = 1)	− 0.101	0.139	0.168
Nursing vignette (Yes = 1)	0.106	0.099	0.285
(3) Confidence			
Constant	2.850	0.379	< 0.001
Maximization	0.010	0.010	0.326
Perceived expertise	0.304	0.075	< 0.001
Gender (Female = 1)	− 0.331	0.137	0.016
Nursing vignette (Yes = 1)	− 0.037	0.097	0.703

Spearman’s correlations were calculated for those participants who answered the four vignettes (*n* = 113). Higher maximization was found to be associated with a greater number of ‘nonaction’ options (H1) (*r* = 0.21, *p* = 0.026), whilst having more years of experience was associated with more ‘immediate action’ responses (*r* = 0.26, *p* = 0.012) and higher perceived expertise (*r* = 0.60, *p* < 0.001). Overall, greater maximization scores significantly predict greater decision time (*p* = 0.034), with no significant relationship with perceived difficulty (H2) or confidence (H5). No other significant correlations existed between maximization scores, years of experience, perceived expertise (H4), and number of each type of decision. Perceived expertise is associated with greater confidence in the decision (H6) (*p* < 0.001). Females reported less confidence in their decision than males (*p* = 0.016). No increase in adjusted R^2^ was found for any of the DVs when the interaction between maximization and type of vignette was included (H3), which reveals lack of moderation effect.

## Discussion

Our study is the first to analyse the possible influence of trait maximization on acute care decision-making by nurses. For this, we designed and presented four vignettes to a sample of 153 experienced Spanish nurses. Based on a review of the literature, we proposed five hypotheses regarding the relationships between maximization, expertise, objective choice behaviour, and subjective choice experience.

As hypothesized in H1, we found that people high in maximization take longer to choose and are more likely to choose non-action, which is consistent with previous findings that maximizers are slower to decide and are more avoidant^22,45^. However, we found no relationship between maximization and the subjective experience of the person making the choice (H2). Contrary to H4, maximization had no significant correlation with years of experience nor perceived expertise. Greater perceived expertise was associated with greater confidence, as suggested in H6, but not with the other indicators of avoidant decision-making, including observable behaviour (H5). Our participants’ responses varied as a function of the type of vignette they were presented—yet no differences were found in the impact of maximization on decision-making, which supports the general-domain view on this trait (H3). When participants answered nursing vignettes, they took longer to respond, but chose more immediate action, and less delayed action than when they answered non-nursing vignettes.

The differences with previous research may be due, at least partly, to some of the study’s features. First, our choice of the MTS-7 was guided by practical reasons, but this instrument represents only one of the possible conceptualisations of the construct maximization, so it may not be the most suitable for explaining its influence on decision-making in this context. In this sense, studying components suggested by other authors but not measured by the MTS-7, such as alternative search or decision difficulty^30^, may shed light on individual differences in this behaviour. Second, it is necessary to consider the limitation implied by the use of a small number of written vignettes. This type of material has been popular in decision making studies for many decades^46^ and our vignettes are based on expert judgment based on real situations experienced by nurses; however, they lack valuable elements like images, videos, and other media that could be used in future works to increase the level of immersion^47^, and they are far from representing the universe of elements that may be present in acute care scenarios. The moderate sample size, as well as the fact that it consists of nurses from a relatively limited geographical area, pose limitations to the generalizability of the results. The fact that one of the authors is a nursing professional with extensive international experience allows for an assumption of validity beyond Spanish borders, but research demonstrates that decision-making is culturally contingent^48^, and social and cultural values influence decision-making processes and styles^49^. Comparative multinational studies, as well as the extension to other healthcare professionals, can help define the generalisability of our findings.

It is reasonable to assume that nurses are especially aware that decision-making in their professional setting—especially in complex and high-risk situations like acute caring—always requires to act thoughtfully and consider a wide number of factors. There is also evidence that, whilst men select the information from the environment that supports their goal-oriented decisions, women tend to use all the information available, weighing benefits and consequences^50^, which may reduce the impact of other possible factors like the personality trait maximization. More than 80% of our participants were women, which does not allow for gender comparisons, but this ratio is a fair representation of the situation of nurses across the world. However, our data show the outcome of decision making, but say little about the process that led to it. In this sense, future work could complement our approach with individual interviews after the response to the vignettes.

Our results have methodological and practical implications. On one hand, our results can be considered consistent with Nibbelink and Reed's^9^ Practice-Primed Decision Model (PPDM) for nursing to the extent that they highlight the importance of variables such as experience and contextual aspects. The model only includes individual differences amongst the variables that contribute to the initiation of the decision-making process through situation awareness—for instance, there is evidence that this aspect is directly related to a person's ability to reason by analogy^51^. Situation awareness was not explored in our research as the process was initiated by the explicit instructions in the vignettes. We are not aware of any study that has addressed the possible impact of maximization or other personality variables on situation awareness, but it represents an interesting line of research for the future.

In practical terms, identifying the role of maximization in decision-making can contribute not only to a better person-task match but also to the well-being of nurses. In this regard, numerous studies reveal that individuals high in maximization express lower life satisfaction, happiness, optimism, and self-esteem^52,53^. The optimization and safeguarding of the well-being of healthcare workers through appropriate organisational and psychological support are part of the obligations of nursing service managers^54^, as well as an obvious social right.

### Supplementary Information

Supplementary Information.

## Data Availability

This study’s data are available on request from the corresponding author. The data are not publicly available due to the restrictions as specified in the Organic Law 3/2018, of December 5, on Protection of Personal Data and Guarantee of Digital Rights Law (Spain). Written Informed consent was obtained from all participants.
