# Supplementary material for: Maximization delays decision-making in acute care nursing
Source: Sci Rep. 2024 Mar 6;14:5482. doi: 10.1038/s41598-024-56037-x (PMC10914817; doi:10.1038/s41598-024-56037-x)
Supplement: Supplementary file 1 — Supplementary Information. [file 41598_2024_56037_MOESM1_ESM.docx]

**Appendix A**

*Vignette 1 – Shop scenario (non-nursing)*

[Context] You are the owner of a small shop in a mid-sized town, where you work with a young assistant. Your business has been closed for 4 months due to the covid lockdown, but the lockdown has been lifted today, and you hope to bounce back after a terrible year.

[Incidence] It is the second week after the lockdown was lifted, and every morning a group of 3-4 suspicious-looking young men occupy a bench right in front of your shop and spend there most of the day, idly smoking, drinking from cans they hide in paper bags, and listening to music from a smartphone. This has been going on for about ten days. As a consequence, your mostly elderly clientele has stopped visiting your shop.

*Vignette 2 – Hiking scenario (non-nursing)*

[Context] Saturday, June, 8:45pm. You are on a country walk with your cousin Olivia (female, 22 years-old, city girl). You walked about 8 miles, had a picnic, and get ready to walk back following the same route; sunset is at 10pm so you are worried it might get really dark before you arrive. You are not familiar with the location, though it is a well-signalled regular footpath. During the day, you have seen some people in the path, but the last one was about two hours ago.

[Incidence] 9:12pm. Olivia slips, has a bad fall, and twists an ankle, which quickly begins to swell; it is very painful, and she is crying. She definitely cannot walk. Your mobile phone has no signal (you recall you lost the signal as soon as you started walking this morning). You suggest going for help whilst she waits, but she is very distressed and does not want to be left alone.

*Vignette 3 – CCTV scenario (nursing)*

[Context] Night shift on a weekend. You are a nurse in a hospital unit with 26 beds for covid patients (24 occupied), distributed in double rooms. There is one assistant supporting you; there should be another nurse, but they are on sick leave, which means you are the only nurse present. Each room has a CCTV system. 2 patients in different rooms require special monitoring: (1) Mr Taylor. Male, 45 years-old, with sudden desaturation (84%) without dyspnoea; he feels ‘nothing’ and is barely conscious. He is on oxygen. Saturation is measured every half hour, with difficulties to maintain 91%-92%; if lower, Mr Taylor is candidate to ICU. (2) Mr Evans. Male, 70 years-old, with increasing disorientation. He has suffered several episodes of acute confusional syndrome with hallucinations. He is on oxygen and saline solution.

[Incident] 4:45am. You are with the assistant in the control room, taking a short break. Suddenly, the assistant calls your attention – both Mr Taylor and Mr Evans seems to be having issues. Mr Taylor’s seems distressed and is making anxious gestures to the camera. Mr Evans, in turn, seems to be going through another episode of confusional syndrome and is fidgeting with the saline solutions and the oxygen supply, apparently trying to pull them out.

*Vignette 4 – CPAP scenario (nursing)*

[Context] Night shift. You are a nurse at a new unit for the close care of critical covid patients; one auxiliary nurse is the only other staff. The unit has 6 individual cubicles fully isolated, each with non-invasive mechanical breathing equipment. All cubicles are occupied. The unit has 3 CPAP (Continuous Positive Airway Pressure) machines, all of them also occupied.

[Incident] 2:51am. The auxiliary nurse just went to the staff room to buy something from the vending machines. As you check on the patients, you realize the condition of two of them has worsened significantly since your checked on them just a few minutes ago: (1) Mr Lewis. Male, 58, with CPAP. He approaches a comatose state with low level of consciousness, hypotension, and low response to pain. The unit does not have appropriate equipment to deal with the hypotension and there is no doctor to intubate him. (2) Mrs Johnson. Female, 38, on high flow therapy. Her oxygen saturation has dropped to 82%-84% and she shows tachypnoea and 39 degrees of temperature. No CPAP is available.
